# Beyond Arabidopsis: BBX Regulators in Crop Plants

**DOI:** 10.3390/ijms22062906

**Published:** 2021-03-12

**Authors:** Urszula Talar, Agnieszka Kiełbowicz-Matuk

**Affiliations:** Institute of Plant Genetics, Polish Academy of Sciences, Strzeszyńska 34, 60-479 Poznań, Poland; utal@igr.poznan.pl

**Keywords:** B-box proteins, transcription factors, growth, development, stress response, crop plants

## Abstract

B-box proteins represent diverse zinc finger transcription factors and regulators forming large families in various plants. A unique domain structure defines them—besides the highly conserved B-box domains, some B-box (BBX) proteins also possess CCT domain and VP motif. Based on the presence of these specific domains, they are mostly classified into five structural groups. The particular members widely differ in structure and fulfill distinct functions in regulating plant growth and development, including seedling photomorphogenesis, the anthocyanins biosynthesis, photoperiodic regulation of flowering, and hormonal pathways. Several BBX proteins are additionally involved in biotic and abiotic stress response. Overexpression of some *BBX* genes stimulates various stress-related genes and enhanced tolerance to different stresses. Moreover, there is evidence of interplay between B-box and the circadian clock mechanism. This review highlights the role of BBX proteins as a part of a broad regulatory network in crop plants, considering their participation in development, physiology, defense, and environmental constraints. A description is also provided of how various BBX regulators involved in stress tolerance were applied in genetic engineering to obtain stress tolerance in transgenic crops.

## 1. Introduction

Intricate regulation of plant growth and development processes depends mainly on precise spatial and temporal control of gene expression mediated by chromatin modifications in reply to endogenous or external stimuli in the environment. Recognition of the transcriptional profile of genes encoding most plant-specific transcription factors and chromatin regulators is fundamental for understanding and elucidating many plant biological processes. Recent studies have demonstrated significant findings regarding B-box (BBX) proteins, representing a diverse group of zinc finger transcription factors and regulators based on their structure and functions.

The *BBX* genes have been present in all eukaryotic genomes analyzed so far, with the highest number of members within all kingdoms. The availability of complete plant genomic sequences has led to the identification of the B-box (*BBX*) gene family, consisting of 64 *BBX* representatives in apple, 37 in white pear, 32 in Arabidopsis, 30 in rice and potato, 29 in tomato, 25 in pear, and 24 in grapevine [1,2,3,4,5,6,7,8]. Regardless of the species, all BBX family members have one single B-box domain or two arranged in tandem, classified into two types, known as B-box1 (B1) and B-box2 (B2), depending on their consensus sequence and the distance between the zinc-binding residues. Some BBX proteins also possess CCT-domain and several conserved motifs localized outside the domains mentioned above [9,10].

In plants, especially in Arabidopsis, the BBX family has been significantly expanded and functionally well-characterized. Considering the importance of crops, the study of BBX proteins in these plants has become more intense. The diverse functions of BBX in plant growth and development range from the involvement in seedling photomorphogenesis [1,10,11,12,13,14,15,16,17], seed germination, photoperiodic regulation of flowering [18,19], and shade avoidance [20,21,22] to responses to biotic and abiotic stresses that have been studied [2,23,24,25,26].

Here, we provide a brief story of the functionally characterized B-box-type zinc finger proteins specific to crop plants and emphasize recent evidence supporting their essential functions in plant development and stress response. This review highlights a crucial role of BBX proteins as part of a broad regulatory network in crops.

## 2. Structural Characteristics of *BBX* Genes

All of the plant BBX proteins have a standard feature, which is a B-box domain. B-box domains are members of zinc finger (ZF) domains, which are some of the most structurally varied among protein domains [27]. Initially, the B-box domain had been classified as a member of the zinc ribbon fold group. However, based on the currently available sequences and structures, it has been transferred to the RING-like treble clef family [28]. It is worth mentioning that there are no reports regarding structural studies carried out on the B-box so far.

### The Function of the B-Box Domains and Conserved Motifs Outside

The structural-level B-box domain has around 40 amino acids in its length, and they fall into two types, 1 and 2. These two types differ in the consensus sequence and the space of the seven or eight Zn(II)-binding residues [29]. The B-box domain has a vital role in protein–protein interaction and mediating transcriptional regulation [30].

Some BBX proteins also have a CCT domain (CONSTANS, CO-LIKE, TIMING OF CAB1: TOC1) [31]. Initially, the presence of a CCT domain was found in CONSTANS (CO), CO-LIKE, and TIMING OF CAB1 (TOC1) proteins in Arabidopsis thaliana, which act as critical flowering regulators. This domain has 42–43 amino acid residues localized at the C-terminus. Comparison of the amino acid sequence of BBX proteins in different plant species revealed that the CCT domain is highly conservative [31]. Many reports have shown that the CCT domain has an essential function in transcriptional regulation [32,33]. The nuclear localization signal (NLS) is a part of the CCT domain and plays a crucial role in locating BBX protein in the nucleus [17].

Besides B-box and CCT domains, Holm et al. [9] discovered a binding sequence motif for BBX protein–protein interaction called the VP motif. The VP (VALINE–PROLINE) motif consists of six amino acids with consensus sequence G-I/VV-P-S/T-F, located at the protein molecule’s carboxy end, separated by 16–20 amino acids from the CCT domain [9].

Additionally, seven new motifs (M1–M7) specific to each structural group were identified [31]. However, their role has not yet been defined, except for the M6 motif, which can significantly impact the functional determination of BBX proteins belonging to the same structural group. The conversion of motive M6 from AtBBX21 to M6 from AtBBX24 has abolished its function in promoting photomorphogenesis [34].

## 3. Look into the Genomes: BBX Family from Arabidopsis to Crops

Arabidopsis, as a model plant, has been extensively investigated for BBX proteins. A transcription factor, CONSTANS (CO), involved in the flowering pathway, was the first identified B-box protein [35]. The B-box family in *Arabidopsis thaliana* consists of 32 proteins. Characterizations of 16 *COL* (*CONSTANS-like*) genes and 8 *DBB* (*Double B-box*) genes by Robson et al. [32] and Kumagai et al. [27], respectively, have made an outstanding contribution in collecting members of the B-box family in this model plant. To provide a uniform nomenclature for the B-box protein family, Khanna et al. [1] published a complete set of all Arabidopsis genes with B-box motifs. Since that report, B-box protein families have been rapidly identified and characterized in other plant species. A genome-wide survey of *BBX* genes considered chromosome localization, gene structures, conserved domains, phylogenetic relationships, subcellular localizations, promoter *cis*-regulatory elements, and expression patterns under the diurnal cycle and stress or hormone treatments.

### 3.1. Classification of BBX Genes in Crops

**Figure 1:**
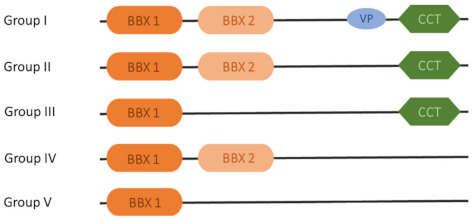
Scheme of B-box (BBX) proteins with main domains in each structure group.

The *BBX* gene family can be divided into five structural groups depending on one or two B-box domains and the CCT domain (Figure 1). The first and second groups consist of proteins with two B-box domains and a CCT domain. Additionally, the VP motif is composed of six amino acid residues, localized in C termini, and has been established as belonging in the first group. In the third group, proteins have one B-box and one CCT domain. The fourth group consists of two tandem B-box domains, and in the fifth group, proteins have a single B-box domain. Crocco and Botto [31] conducted a comprehensive evolutionary analysis of the BBX protein family in 12 plant species that started from green algae and ended with dicots. The results showed that each of the five BBX protein groups evolved independently during plant evolution. Some literature has distinguished BBX proteins in separate subfamilies, including *COL* (CO-like) and *DBB* (double B-box). Indeed, the *COL* family contains proteins with double B-box and CCT domains, and they are homologs of the CONSTANS protein. The *DBB* proteins lack the CCT domain and have two tandem-localized B-box domains in the sequence.

#### 3.1.1. Cereal Crops

Rice was the first crop to have the whole B-box protein family identified [2]. In this plant, 30 *OsBBX* genes have been identified and named according to rice chromosomes position. *OsBBX* genes are distributed in all chromosomes, omitting chromosomes 10 and 11 (Table 1). A segmental duplication analysis showed that 18 *OsBBX*s are located in the chromosomes duplicated segmental regions [2].

Maize (*Zea mays*) includes 19 *COL* [36] and 12 *DBB* [37] genes in its genome. All *BBX* genes are distributed on 9 of 10 maize chromosomes, except chromosome 8, and their nomenclature refers to their position on chromosomes (Table 1). Eight *ZmCOL* gene pairs have been identified to be involved in segmental duplications. Simultaneously, the expansion of the maize *ZmDBB* gene family occurred at the same duplication event.

#### 3.1.2. Rosaceae Species

According to phylogenetic relationships and domains, 64 *BBX* genes in the apple genome are divided into five groups. This number of genes is significantly large compared to *BBX* genes in other plants, and it suggests that tandem, segmental, or genome-wide duplication in apple might cause this phenomenon [6]. A total of 50 genes have been mapped into 15 of 17 apple chromosomes (Table 1). Identifying the chromosome position for the remaining 14 genes was not successful, probably caused by incorrect assembling of genomic sequences.

In the pear genome (*Pyrus bretschneideri* Rehd.), 25 *BBX* genes have been identified, clustered in five groups, and sequentially named [5]. All the *PbBBX* genes are distributed among 12 of the total 17 pear chromosomes. The presence of segmental duplication for 13 gene pairs and no single tandem duplication is characteristic of *BBX* genes in this species. By contrast, in other Pyrus species, *Pyrus pyrifolia*, a total of 39 *BBX* family members were identified and were named according to the chromosomal distribution [38].

Shalmani et al. [39] have recently identified the *BBX* gene family in other *Rosaceae* species like rose, peach, strawberry, sweet cherry, and black raspberry with 22, 20, 21, 22, and 20 *BBX* members, respectively.

#### 3.1.3. Solanaceae Species

In tomato, 29 putative *BBX* genes have been identified and named according to their homology to Arabidopsis *BBX* genes. The whole family is distributed within all chromosomes except for chromosome 11. The nuclear location of most tomato BBX proteins have been envisaged using in silico analysis, and has been confirmed for seven of them by Arabidopsis mesophyll protoplast assay [3].

A comparable number of *BBX* genes, 30, have been discovered in potato, and numbered based on BBX and CCT domains length and presence. Except for chromosome 11, potato *StBBX* genes are widely distributed in the whole genome [4].

#### 3.1.4. Other Crops

In cotton, 42 *GhCOL* genes were identified in the genome, distributed unevenly along 18 different chromosomes. Phylogenetic analysis clustered them into three groups, whereby 14 *COL* genes in group I showed conserved structure compared with other plants. Analysis of gene expression patterns in group I concluded that these genes are potentially involved in photoperiodic flowering and light signaling regulation [40].

Comprehensive bioinformatics analysis of whole genomes of grapevines led to the detection of 24 *BBX* genes, of which 22 genes are evenly distributed in 11 of the 19 chromosomes, while the two genes are not assigned to any position [8].

The same number of *BBX* genes, 24, were detected on nine of the ten chromosomes in a wild peanut [41].

In bananas, 25 *COL* genes belong to group I–III. Nine genes from group I were investigated by Chaurasia et al. and the results showed that those genes are highly conserved in structure compared to members in other plants [42].

In soybeans, 26 *CO*-like genes are classified into three clades, comprising 13 homologous pairs [43]. On the contrary, only 17 putative *COL* genes were identified in leek, a herbaceous plant belonging to the *Amaryllidaceae f*amily [44]. Four of these leek *COL* genes show high sequence similarity with key factors modulating the heading date in barley and rice.

Sugar beets have been demonstrated to possess at least 10 *CONSTANS-LIKE* genes. However, these data are based on ESTs collection availability, whereas the sugar beet genome sequence was published a few years after that [45]. Therefore, it is expected that a larger number of genes may be identified.

## 4. Time to Switch from Vegetative to Generative Development

Strict regulation of flowering time is essential for plant reproductive success, enabling seed development completion in beneficial environmental conditions [46]. The photoperiodic flowering induction mechanism has been best recognized and characterized in *Arabidopsis thaliana*, where the FLOWERING LOCUS T (FT) and the CONSTANS (CO/BBX1) are the critical elements [47,48,49]. Research has shown that the AtBBX1, the first identified and characterized protein belonging to the BBX family, plays an essential role in regulating flowering time and flower development [19]. Besides, several other proteins belonging to the BBX family also perform a crucial role in regulatory networks, controlling floral transition and flower formation in Arabidopsis, including AtBBX4/COL3 [50], AtBX6/COL5 [51], AtBBX7/COL9 [52], AtBBX10/COL12 [53], and AtBBX17/COL8 [54].

**Figure 2:**
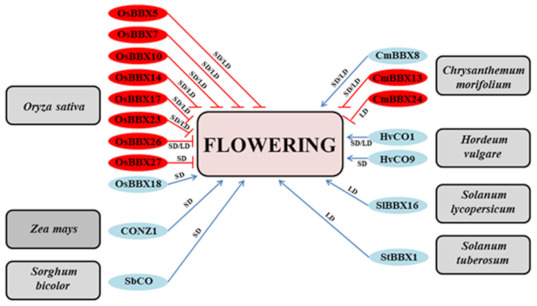
Involvement of B-box proteins as positive (blue) and negative (red) regulators of flowering in various crop plants including *O. sativa*: OsBBX5 (ID: Os02g0610500) [56], OsBBX7 (ID: Os02g0724000) [57], OsBBX10 (ID: Os03g0711100) [58], OsBBX14 (ID: Os05g0204600) [67], OsBBX17 (ID: Os06g0264200) [68], OsBBX18 (Os06g0275000) [55], OsBBX23 (ID: Os07g0667300) [59], OsBBX26 (ID: Os08g0536300) [60], OsBBX27 (ID: Os09g0240200) [69]; *Z. mays*: CONZ1 (ID: ABW82153) [61]; *S. bicolor*: SbCO (ID: Sb10g010050) [63]; *C. morifolium*: CmBBX24 (ID: KF385866) [64], CmBBX13 (ID: KP963935) [70], CmBBX8 (ID: KP96393) [71]; *H. vulgare*: HvCO1 (ID: AF490467) [65], HvCO9 (ID: AY082965) [66]; *S. lycopersicum*: SlBBX16 (ID: Solyc12g005750.1) [72]; *S. tuberosum* (ID: AM888389.1) [73]. LD—long day, SD—short day.

Undoubtedly, less is known about the function of BBX proteins in crop growth and development. However, many BBX proteins in plants other than Arabidopsis are also likely to play a role in these processes. In rice, the short day (SD) plant, the GI–CO–FT regulatory pathway is conserved and flowering time is mutually regulated by two different photoperiodic pathways, in which several BBX members act as flowering inductors or repressors (Figure 2). The rice CO ortholog, Hd1 (HEADING DATE 1)/OsBBX18, promotes flowering under inductive short-day conditions by regulating the *Heading date 3a* (*Hd3a*) and *Rice FT-like 1* (*RFT1*) florigen genes [55]. Hd3a is also induced by another flowering activator, Ehd1 (Early heading date 1), which functions independently of Hd1 under SD conditions. Meanwhile, under noninductive long-day conditions, Hd1 turns into a flowering repressor and affects the expression of *Hd3a*. Another key repressor of flowering in rice is a small protein termed Ghd7 (Grain number, plant height, and heading date 7), which acts as an LD-specific repressor of *EHd1* expression. So far, other proteins belonging to the BBX family in rice that may negatively affect flowering under two different photoperiodic conditions have been identified (Figure 2). Among them, OsBBX5(OsCOL4), OsBBX7(OsCOL9)*,* OsBBX10(OsCOL10), and OsBBX23(OsCOL13) repress flowering by reducing the expression of *FT-*like genes and heading date through *Ehd1* (Early heading date 1) [56,57,58,59]. Moreover, some BBX proteins, including OsBBX10(OsCOL10) and OsBBX26(OsCOL15), act downstream of Ghd7 repressor, reducing expression of *Ehd1* [58,59,60]. In maize, a typical SD plant, the most critical period in the whole development is the flowering time that determines the size of the cob formed by the plant and its filling with grain to a significant extent. In this species, the B-box-type gene corresponding to the Arabidopsis *CO*, called *Conz1*, activates the *FT*-like *ZCN8,* which functions as a floral inductor involved in photoperiod sensitivity in maize [61,62]. The *AtCO* gene homologs, *SbHd1, HvCO1*, and *HvCO9,* have also been found in other cereal crops, such as sorghum and barley, respectively, representing the long-day (LD) plants [63,64,65]. Under LD conditions, SbHd1 activates flowering by inducing *SbCN8* and *SbCN12* (orthologs of maize *ZCN8* and *ZCN12*, respectively) [63], while HvCO1 and HvCO9 are involved in the activation of *FT*-like genes required for flowering induction in barley [65,66].

AtCO homologs in different species, including potato [74], ryegrass [75], grape [76], and alfalfa [77], are also presumably involved in photoperiodic flowering induction. Additionally, in potatoes, *StCO* regulates photoperiodic tuberization in a graft-transmissible manner [78]. The genes corresponding to the tomato *FT* homolog, designated *StSP6A* and *StSPD3,* have been identified in wild potato species *S. tuberosum ssp.* *andigena* [73,79,80]. As shown, the *StSP6A* encodes a protein promoting tuber formation [73], while *StSPD3* encodes a protein promoting floral development [79,80]. Potato StCOL1 (BBX1) protein controls *StSP6A* expression through direct activation of an additional *FT* family member, while *StSP5G*, which acts as a repressor of *StSP6A i*n leaves, mediates the strict short-day (SD) requirement of *andigena* plants for tuberization [73].

Other *BBX* genes, apart from *BBX1/CO*, are implicated in regulating flowering time in crops as well. In tomato *Sl*BBX16, the closest homolog of Arabidopsis microprotein 1b interacts with TCMP-2, a small family member of tomato cystine-knot proteins, and affects tomato flowering [81]. Arabidopsis plants ectopically overexpressing the *TCMP-2* exhibited an increased level of FLOWERING LOCUS T (FT) mRNA and anticipated flowering. Previously, the same authors revealed that transgenic tomato plants *pTCMP-2::TCMP-1* with increased *TCMP-2* expression in flower buds showed accelerated termination of the sympodial units [72]. In chrysanthemum, three B-box proteins have been identified so far that play a positive or negative role in regulating flowering time. One of them, CmBBX8, stimulates flowering in summer-flowering chrysanthemum grown under LD conditions [71], while CmBBX13 and CmBBX24 proteins cause late flowering under long- and/or short-day conditions [64,65,66,70,72,73,74,75,76,77,78,79,80,81].

Understanding the flowering mechanisms and the role of B-box proteins in the photoperiodic flowering pathways in various crops is a crucial interest. Although the regulatory network triggering flowering is conserved in many species, the function of BBX acting downstream of the photoperiod response to accelerate or prevent floral initiation may vary significantly among plants.

## 5. Crops *BBX* Genes in the Anthocyanins Biosynthesis

Anthocyanins are pigments responsible for the red to black color of plant organs, such as flowers or fruits. Besides visual effects and market value, the accumulation of anthocyanins in organs is connected with biotic or abiotic stress, such as viral pathogens, wounding, or drought [82,83]. Those pigments also take part in protection against photo-oxidative and heat damage [84]. A few apple BBX proteins, including MdBBX1, MdBBX20, and MdBBX33/MdCOL11 are regulator factors of anthocyanin synthesis. MdBBX33 is a close homolog of Arabidopsis AtBBX22, and its overexpression causes an increased anthocyanin level in Arabidopsis seedlings [85]. MdBBX33 protein regulates anthocyanin accumulation, influencing the red skin color of apples in a light and temperature-dependent manner. Both low temperature and UV-B light correlate with upregulation of *MdBBX33* expression and positively affect anthocyanin accumulation in apple fruits [85]. Furthermore, the expression of two anthocyanin accumulation-responsible genes, *MdMYBA* and *MdbHLH*, increase in the fruit ripening stage, which is associated with the increase of *MdBBX33* transcript level.

On the contrary, another *BBX* gene, *MdBBX1*, when overexpressed in apple, does not directly increase anthocyanin accumulation. However, MdBBX1 may activate two essential genes—an MYB activator, *MYB10,* and the anthocyanin biosynthetic gene *DRF (DIHYDROFLAVONOL 4-REDUCTASE)—*by binding to a CCAAT motif present in their promoter region*,* ultimately leading to increased anthocyanin levels [86]. Moreover, expression patterns of some other *BBX* genes in apples, such as *MdBBX15*, *MdBBX17, MdBBX35*, *MdBBX51*, and *MdBBX54*, are correlated with anthocyanin induction in apple fruit skin [86]. Transactivation assays on the *MYB10* promoter revealed that these BBX proteins could function as activators via direct induction of the apple anthocyanin-regulating MYB10 [86].

Studies on apples revealed that ultraviolet treatments promote some BBX transcription factors, which activate the expression of main anthocyanin biosynthetic genes and ultimately lead to increased anthocyanin levels. One of them is MdBBX20, which stimulates anthocyanin accumulation under ultraviolet radiation and low-temperature conditions. Overexpression of *MdBBX20* caused increased anthocyanin accumulation in transformed calli [87]. Furthermore, MdBBX20 interacts by its B-box2 domain with transcription factor HY5, and in complexes, it regulates transcription of *MdMYB1/MdMYB10,* the anthocyanin key regulator concentrations, by binding its G-box *cis*-element. MdBBX22 is another UV-inducible protein that directly interacts with MdHY5 and enhances the binding to key anthocyanin synthesis factors, *MdMYB10* and *MdCHS*. Overexpression of *MdBBX22* has been shown to induce anthocyanin biosynthesis [88,89]. Interestingly, MdBBX24, MdBBX33, MdBBX37, and MdBBX48 also interact with MdHY5, suggesting that numerous BBX might be entangled with anthocyanins synthesis [88,89].

It is also worth mentioning that some BBX members in apples, including MdBBX20, MdBBX22, MdBBX23 MdBBX24 MdBBX25 MdBBX33, and MdBBX43, interact with MdBT2 protein, known as a negative regulator of the UV-B-induced anthocyanin biosynthesis. An et al. [88] revealed that MdBT2 degrades MdBBX22 protein through the 26S proteasome pathway and the other members of the BBX family might be ubiquitination substrates for MdBT2.

So far, two BBX proteins have been identified that act as positive regulators of anthocyanin accumulation in a red pear. One of them is nuclear-localized protein, PpBBX16, a close homolog of AtBBX22, that favorably controls anthocyanin production in light-induced conditions via activating *PpMYB10* [38]. However, PpBBX16 cannot directly bind the promoter of *PpMYB10* and requires the presence of PpHY5 to achieve complete functionality. Moreover, PpBBX16 can promote the expression level of anthocyanin-related genes, such as *PpCHI*, *PpCHS*, and *PpDFR*, as was shown in the dual-luciferase assay introduced in tobacco. Overexpression of *PpBBX16* in Arabidopsis seedlings increased anthocyanin content in the hypocotyls and tops of flower stalks. Furthermore, other BBX protein PpBBX18 also physically interacted with PpHY5, thus inducing transcription of *PpMYB10* and consequently regulating anthocyanin biosynthesis in Arabidopsis and pear [38].

Besides positive regulators of anthocyanins biosynthesis, BBXs also play a role as negative regulators. In apples, MdBBX37 was indicated as an inhibitor of anthocyanin biosynthesis. Its interactions with pivotal positive regulators MdMYB1 and MdMYB9 block the binding to their target genes. Also, it acts as a suppressor of *MdHY5* expression by binding to its promoter [88,89]. Meanwhile, in pears, PpBBX21 protein directly interacts with PpBBX18 or PpHY5, inhibits PpBBX18-PpHY5 complex formation, and represses anthocyanin biosynthesis [90].

BBX proteins are involved in the precise control of anthocyanin synthesis by binding to HY5 and transcriptional regulation of *MYB10.* Likewise, modulation of expression of other essential genes involved in anthocyanin production provides new insights into the multifunctionality of these factors. However, many questions remain to be answered to fill the knowledge gaps on light-induced anthocyanin biosynthesis.

## 6. Involvement of the BBX Proteins in Stress Response and Hormonal Pathways

Many reports have indicated that BBX proteins are involved in the signaling pathway induced by abiotic stresses, including low temperature, high salinity, drought, and heat. Some BBX proteins might be engaged in responses to several abiotic stress factors. In Arabidopsis, AtBBX18 negatively regulates thermotolerance through modulation of the expression of heat-stress-responsive genes, such as *DGD1*, *Hsp70*, *Hsp101*, and *APX2* [25]. Another Arabidopsis B-box protein, AtBBX24/STO, enhances the growth of roots in high salt conditions [24]. Regulation of gene expression at the transcriptional level is mostly mediated by sequence-specific binding of transcription factors to the *cis*-acting promoter elements. Numerous *BBX* genes contain several putative stress-related *cis*-acting elements, such as MBS, ARE, LTR, and HSE. The transcript level of many *BBX* genes are altered under different stress conditions, as shown by transcription profiling (Table 2). Thus BBX proteins seem to be essential factors that integrate various signal transduction pathways, replying to diverse stresses and engaging in many cellular processes. However, only a few BBX proteins have been proven to be associated with responses to stress factors so far.

Plants in nature are also exposed to biotic stresses covering a broad spectrum of plant pathogens. Present knowledge indicates that BBX regulators may also participate in the control of plant defense responses. Unfortunately, the understanding of the role of BBX proteins in this process is still in its infancy. The expression of a rice gene, *OsCOL9*, encoding a BBX protein belonging to group II of the COL protein family, has been shown to be enhanced at the mRNA level after *Magnaporthe oryzae* infection. Moreover, transgenic *OsCOL9* knock-out rice plants showed increased pathogen susceptibility [97]. The expression of a banana gene, *MaCOL1*, increased after infection by *Colletotrichum musae* [95]. Overexpression of *IbBBX24* gene significantly increased *Fusarium* wilt disease resistance in cultivated sweet potatoes [96].

Some BBX family members also play essential roles in hormone signaling pathways. There are many reports documenting the response of Arabidopsis *BBX* genes to plant hormones and the involvement of these proteins in many hormonal pathways [98]. Moreover, the transcript accumulation of several *BBX* genes in crops is elevated in response to exogenous treatment of phytohormones, including ABA, GA, JA, and SA (Table 2). Most of these genes possess one or more well-defined hormone-responsive elements in their promoter sequences, like ABRE (ABA—responsive element), ERE (ethylene responsive element), CGTCA-motif and TGACG-motif (MeJA responsive elements), which respond to different hormonal pathways [2,3]. Interestingly, in bananas, MaCOL1 protein can mediate cross-talk between signaling pathways in response to biotic and abiotic stresses since the accumulation of *MaCOL1* transcript was enhanced by chilling and pathogen infection [95].

Thus, transcriptomic analyses using macro- and microarray approaches are excellent tools for identifying new genes related to plant responses to different stresses and exogenous hormone treatments. However, the alternations in gene expression are frequently not reflected at the protein level. Therefore, the dynamic coordination of transcription seems essential to verify observed changes in expression profiles in response to external and internal signals.

## 7. Stress Response of Transgenic Plants Overexpressing the *BBX* Regulators

Recognition of plants’ genetic and molecular resistance mechanisms to environmental stimuli allows researchers to design the new strategies to improve plants’ stress tolerance. Although abiotic stress tolerance is a polygenic trait, single genes encoding crucial transcriptional regulators can improve plant adaptation to various stresses by turning regulatory gene networks on and off. The significance of some BBX proteins in stress tolerance has been revealed by manipulating the genes encoding such proteins in transgenic economically essential plants to obtain desirable agronomic characteristics and stress resistance. Many studies revealed the potential of manipulating *BBX* genes to confer enhanced tolerance to various stresses. Changing the *BBX* gene expression enhanced stress tolerance in Arabidopsis, chrysanthemums, apples, and rice (Table 3) [64,91,92,94,99,100].

Although analyses of *BBX* gene overexpression in response to defined stress are very informative, studies focusing on crop productivity will provide answers regarding the transgenic plants’ improvements in stress tolerance and yield under field conditions.

## 8. The Interplay between BBX Proteins and the Circadian Clock

Most processes in living organisms evolve cyclically. The rhythmic course of phenomena is the result of organisms’ adaptation to periodically changing conditions on Earth. In plants, the synchronization of the development cycle with cyclical changes in the environment is possible by developing an endogenous mechanism of the biological clock, which generates rhythms of a ~24 h period [102]. Since the expression of numerous genes in crops is controlled at the transcript level by the biological clock, it indicates that the circadian oscillator affects agricultural importance traits. Several oscillator components have been identified as essential determinants of yield-related traits [103].

Many genes, the expressions of which are controlled by the biological clock, encode proteins containing the B-box zinc finger domain [2,4,26,27]. In fact, in Arabidopsis, some BBX proteins involved in flowering are under circadian clock control. Thus the expressions of AtBBX1/CONSTANS and AtBBX32 are regulated by the biological clock [27,50]. Moreover, transcriptional analysis of other *BBX* genes in Arabidopsis revealed circadian*-*dependent regulation of *AtBBX18*, *AtBBX19, AtBBX22*, *AtBBX24,* and *AtBBX25* [17,27]. In the promoter regions of clock controlled genes, the specific *cis*-elements “CAANNNATC” associated with the circadian regulation were found [4,104]. The transcription factor StZPR1, belonging to the zinc finger family type C_4_, has been identified recently, which binds to the “CAACAGCATC” motive defined by the term CIRC (circadian regulated) in the *StBBX24* gene promoter in *Solanum tuberosum*. Moreover, in potato transgenic plants with silenced *StZPR1* expression*,* there are disturbances of some *BBX* genes daily oscillations, such as *StBBX5*, *StBBX9*, *StBBX18*, *StBBX24* and *StBBX27* [105]. It is also noteworthy that the circadian clock is able to interrupt an effect of external stimuli on some *BBX* expressions. This interruption allows plants perform temporal gating in response to environmental constraints, thus triggering appropriate reactions for stress at a more suitable time of a day [26].

The full extent of the mechanisms by which plant keep the clock is still under investigation. Moreover, understanding the multiplatform link between the clock genes and cell-level circadian responses involving large *BBX* gene networks remains unexplained.

## 9. Summary and Prospects

BBX proteins constitute a complex regulatory network in planta [17,21,98,106]. Despite considerable progress in understanding B-box proteins’ function in growth and development and stress responses in crops, the physiological role and the molecular mechanisms for many of them remain still unknown. Knowledge of protein partners for B-box proteins under different circadian cycles and environmental conditions and identifying critical regulators of their transcription will provide insight into molecular relationships between structure and function of this family. More information regarding the functions of BBX might help to understand the complexity of signaling pathways generated by the biological clock. However, to provide new insights into the role of BBX proteins in plants, more time-consuming experimental in vivo data, as gene overexpression and knock-outs, are required.

## Figures and Tables

**Table 1:** *BBX* gene distribution on crop plants chromosomes.

Plant Species	No. of *BBX* Genes	No. of Chromosomes Having *BBX*/No. of Plant Chromosomes	Chromosome with no *BBX*	References
Rice (*Oryza sativa*)	30	10/12	10 and 11	[2]
Maize (*Zea mays*)	31	9/10	8	[36,37]
Apple (*Malus domestica*)	64	15/17	4 and 15	[6]
Pear (*Pyrus bretschneideri*)	25	12/17	1,2,4,7,12	[5]
Pear (*Pyrus pyrifolia*)	39	nd/17	nd	[38]
Rose (*Rosa chinensis*)	22	6/7	1	[39]
Peach (*Prunus persica*)	20	6/8	3 and 6	[39]
Strawberry (*Fragaria vesca*)	21	6/7	7	[39]
Sweet cherry (*Prunus avium*)	22	6/8	2 and 6	[39]
Black raspberry (*Rubus occidentalis*)	20	6/7	7	[39]
Tomato (*Solanum lycopersicum*)	29	11/12	11	[3]
Potato (*Solanum tuberosum*)	30	10/12	11	[4]
Cotton (*Gossypium hirsutum*)	42	18/18	-	[40]
Grapevine (*Vitis vinifera*)	24	11/19	2,6,8,10,13,15,16,17	[8]
Wild peanut (*Arachis duranensis*)	24	9/10	2	[41]
Banana (*Musa acuminata*) *	25	7/11	4,5,6,8	[42]
Soyabean (*Glycine max*) *	26	nd/10	nd	[43]
Leek (*Allium porrum*) *	17	nd/8	nd	[44]
Sugar beet (*Beta vulgaris*) *	10	nd/9	nd	[45]

* only *COL* genes; nd—no data available.

**Table 2:** *BBX* genes in various crop species and their known transcript positive response to various abiotic stresses and hormones.

Gene	Accession No./ID	Response to Abiotic Stress and Hormones									Species	References
		Cold/ Chilling	Drought	Salt	Dehydration	Heat	ABA	GA	SA	MeJA		
*VvZFPL*	HQ179976	+									*Vitis vinifera*	[91]
*SsBBX24*	ABC25454	+		+							*Solanum sogarandinum*	[26]
*MdBBX10*	MDP0000733075			+							*Malus domestica*	[92]
*OsBBX1*	Os01g0202500	+	+	+							*Oryza sativa*	[93]
*OsBBX2*	Os02g0176000	+	+	+				+	+	+	*Oryza sativa*	[93]
*OsBBX7*	Os02g0724000							+	+	+	*Oryza sativa*	[93]
*OsBBX8*	Os02g0731700	+	+	+							*Oryza sativa*	[93,94]
*OsBBX14*	Os05g0204600						+				*Oryza sativa*	[93]
*OsBBX17*	Os06g0264200							+	+	+	*Oryza sativa*	[93]
*OsBBX19*	Os06g0298200	+	+	+				+	+	+	*Oryza sativa*	[93]
*OsBBX24*	Os08g0178800	+	+	+				+	+	+	*Oryza sativa*	[93]
SlBBX1 *	Solyc02g089520.1	+									*Solanum lycopersicum*	[3]
SlBBX7 **	Solyc12g006240.1					+					*Solanum lycopersicum*	[3]
SlBBX16 ***	Solyc12g005750.1			+							*Solanum lycopersicum*	[3]
*MaCOL1*	JQ314345	+									*Musa nana*	[95]
*Cm-BBX24*	KF385866	+			+						*Chrysanthemum* *morifolium*	[64]
*IbBBX24*	MH813941									+	*Ipomoea batatas*	[96]

*Solanum lycopersicum BBX* genes with the same type of responses are marked with asterisks: * *SlBBX3* (Solyc02g089540.2), * *SlBBX9* (Solyc07g045180.2), * *SlBBX19* (Solyc01g110370.2), * *SlBBX21* (Solyc04g081020.2), * *SlBBX28* (Solyc12g005660.1), * *SlBBX29* (Solyc02g079430.2); ** *SlBBX11* (Solyc09g0074560.2), ** *SlBBX12* (Solyc05g024010.2), ** *SlBBX15* (Solyc05g009310.2); *** *SlBBX17* (Solyc07g062620.1), *** *SlBBX18* (Solyc02g084420.2), *** *SlBBX24* (Solyc06g073180.2).

**Table 3:** Stress response of transgenic plants overexpressing *BBX* genes.

Gene	Accession No./ID	From	To	Phenotypes	References
*MdBBX10*	MDP0000733075	*Malus domestica*	*Arabidopsis thaliana*	Salt and drought tolerance	[92]
*VpSBP16*	nd	*Vitis pseudoreticulata*	*Arabidopsis thaliana*	Salt and drought tolerance	[99]
*Cm-BBX24*	KF385866	*Chrysanthemum morifolium*	*Chrysanthemum morifolium*	Tolerance to freezing and drought	[64]
*VvZFPL*	HQ179976	*Vitis vinifera*	*Arabidopsis thaliana*	Cold tolerance	[91]
*AtBBX29*	At5g54470	*Arabidopsis thaliana*	*Saccharum*	Drought tolerance	[101]
*MdBBX37*	MDP0000157816	*Malus hupehensis*	*Malus hupehensis*	Cold tolerance	[100]
*Ghd2(OsBBX8)*	Os02g0731700	*Oryza sativa*	*Oryza sativa*	Drought tolerance	[94]

nd—no data available.

## References

[1] Khanna R., Kronmiller B., Maszle D.R., Coupland G., Holm M., Mizuno T., Wu S.H. (2009). The Arabidopsis B-box zinc finger family. Plant Cell.

[2] Huang J., Zhao X., Weng X., Wang L., Xie W. (2012). The rice B-box zinc finger gene family: Genomic identification, characterization, expression profiling and diurnal analysis. PLoS ONE.

[3] Chu Z., Wang X., Li Y., Yu H., Li J., Lu Y., LI H., Ouyang B. (2016). Genomic organization, phylogenetic and expression analysis of the B-box gene family in tomato. Front. Plant Sci..

[4] Talar U., Kiełbowicz-Matuk A., Czarnecka J., Rorat T. (2017). Genome-wide survey of B-box proteins in potato (*Solanum tuberosum*) – identification, characterization and expression patterns during diurnal cycle, etiolation and de-etiolation. PLoS ONE.

[5] Cao Y., Han Y., Meng D., Li D., Jiao C., Jin Q., Lin Y., Cai Y. (2017). B-BOX genes: Genome-wide identification, evolution and their contribution to pollen growth in pear (*Pyrus bretschneideri* Rehd.). BMC Plant Biol..

[6] Liu X., Li R., Dai Y., Chen X., Wang X. (2017). Genome-wide identification and expression analysis of the B-box gene family in the apple (*Malus domestica* Borkh.) genome. Mol. Genet. Genomics.

[7] Zou Z., Wang R., Wang R., Yang S., Yang Y. (2017). Genome-wide identification, phylogenetic analysis, and expression profiling of the BBX family genes in pear. J. Hort. Sci. Biotechnol..

[8] Wei H., Wang P., Chen J., Li C., Wang Y., Yuan Y., Fang J., Leng X. (2020). Genome-wide identification and analysis of B-BOX gene family in grapevine reveal its potential functions in berry development. BMC Plant Biol..

[9] Holm M., Hardtke C.S., Gaudet R., Deng X.W. (2001). Identification of a structural motif that confers specific interaction with the WD40 repeat domain of *Arabidopsis* COP1. EMBO J..

[10] Datta S., Hettiarachchi G.H., Deng X.W., Holm M. (2006). Arabidopsis CONSTANS- LIKE3 is a positive regulator of red light signaling and root growth. Plant Cell.

[11] Datta S., Hettiarachchi C., Johansson H., Holm M. (2007). SALT TOLERANCE HOMOLOG2, a B-box protein in *Arabidopsis* that activates transcription and positively regulates light-mediated development. Plant Cell.

[12] Datta S., Johansson H., Hettiarachchi C., Irigoyen M.L., Desai M., Rubio V., Holm M. (2008). LZF1/SALT TOLERANCE HOMOLOG3, an Arabidopsis B-box protein involved in light-dependent development and gene expression, undergoes COP1 mediated ubiquitination. Plant Cell.

[13] Indorf M., Cordero J., Neuhaus G., Rodríguez-Franco M. (2007). Salt tolerance (STO), a stress-related protein, has a major role in light signalling. Plant J..

[14] Chang C.S., Li Y.H., Chen L.T., Chen W., Hsieh W.P., Shin J., Jane W.N., Chou S.J., Choi G., Hu J.M. (2008). LZF1, a HY5-regulated transcriptional factor, functions in Arabidopsis de-etiolation. Plant J..

[15] Holtan H.E., Bandong S., Marion C.M., Adam L., Tiwari S., Shen Y., Maloof J.N., Maszle D.R., Ohto M., Preuss A. (2011). BBX32, an Arabidopsis B-Box protein, functions in light signaling by suppressing HY5-regulated gene expression and interacting with STH2/BBX21. Plant Physiol..

[16] Fan X.Y., Sun Y., Cao D.M., Bai M.Y., Luo X.M., Yang H.J., Wei C.Q., Zhu S.W., Sun Y., Chong K. (2012). BZS1, a B-box protein, promotes photomorphogenesis downstream of both brassinosteroid and light signaling pathways. Mol. Plant.

[17] Gangappa S.N., Botto J.F. (2014). The BBX family of plant transcription factors. Trends Plant Sci..

[18] Wenkel S., Turck F., Singer K., Gissot L., Gourrierec J.L., Samach A., Coupland G. (2006). CONSTANS and the CCAAT box binding complex share a functionally important domain and interact to regulate flowering of Arabidopsis. Plant Cell.

[19] Valverde F. (2011). CONSTANS and the evolutionary origin of photoperiodic timing of flowering. J. Exp. Bot..

[20] Crocco C.D., Holm M., Yanovsky M.J., Botto J.F. (2010). AtBBX21 and COP1 genetically interact in the regulation of shade avoidance. Plant J..

[21] Crocco C.D., Holm M., Yanovsky M.J., Botto J.F. (2011). Function of B-BOX under shade. Plant Signal. Behav..

[22] Gangappa S.N., Crocco C.D., Johansson H., Datta S., Hettiarachchi C., Holm M., Botto J.F. (2013). The Arabidopsis B-box protein BBX25 interacts with HY5, negatively regulating BBX22 expression to suppress seedling photomorphogenesis. Plant Cell.

[23] Lippuner V., Cyert M.S., Gasser C.S. (1996). Two classes of plant cDNA clones differentially complement yeast calcineurin mutants and increase salt tolerance of wild-type yeast. J. Biol. Chem..

[24] Nagaoka S., Takano T. (2003). Salt tolerance-related protein STO binds to a Myb transcription factor homologue and confers salt tolerance in Arabidopsis. J. Exp. Bot..

[25] Wang Q., Tu X., Zhang J., Chen X., Rao L. (2013). Heat stress-induced BBX18 negatively regulates the thermos-tolerance in Arabidopsis. Mol. Biol. Rep..

[26] Kiełbowicz-Matuk A., Rey P., Rorat T. (2014). Interplay between circadian rhythm, time of the day and osmotic stress constraints in the regulation of the expression of a Solanum Double B-box gene. Ann. Bot..

[27] Kumagai T., Ito S., Nakamichi N., Niwa Y., Murakami M., Yamashino T., Mizuno T. (2008). The common function of a novel subfamily of B-box zinc finger proteins with reference to circadian-associated events in *Arabidopsis thaliana*. Biosci. Biotechnol. Biochem..

[28] Kaur G., Subramanian S. (2016). Classification of the treble CLEF zinc finger: Noteworthy lessons for structure and function evolution. Sci. Rep..

[29] Kluska K., Adamczyk J., Krężel A. (2018). Metal binding properties, stability and reactivity of zinc fingers. Coord. Chem. Rev..

[30] Qi Q., Gibson A., Fu X., Zheng M., Kuehn R., Wang Y., Wang Y., Navarro S., Morrell J.A., Jiang D. (2012). Involvement of the N-terminal B-box domain of Arabidopsis BBX32 protein in interaction with soybean BBX62 protein. J. Biol. Chem..

[31] Crocco C.D., Botto J.F. (2013). BBX proteins in green plants: Insights into their evolution, structure feature and functional diversification. Gene.

[32] Robson F., Costa M.M., Hepworth S.R., Vizir I., Pineiro M., Reeves P.H., Putterill J., Coupland G. (2001). Functional importance of conserved domains in the flowering-time gene CONSTANS demonstrated by analysis of mutant alleles and transgenic plants. Plant J..

[33] Gendron J.M., Pruneda-Paz J.L., Doherty C.J., Gross A.M., Kang S.E., Kay S.A. (2012). Arabidopsis circadian clock protein, TOC1, is a DNA-binding transcription factor. Proc. Natl. Acad. Sci. USA.

[34] Job N., Yadukrishnan P., Bursch K., Datta S., Johansson H. (2018). Two B-box proteins regulate photomorphogenesis by oppositely modulating HY5 through their diverse C-terminal domains. Plant Physiol..

[35] Putterill J., Robson F., Lee K., Simon R., Coupland G. (1995). The CONSTANS gene of Arabidopsis promotes flowering and encodes a protein showing similarities to zinc finger transcription factors. Cell.

[36] Song N., Xu Z., Wang J., Qin Q., Jiang H., Si W., Li X. (2018). Genome-wide analysis of maize CONSTANS-LIKE gene family and expression profiling under light/dark and abscisic acid treatment. Gene.

[37] Li W., Wang J., Sun Q., Li W., Yu Y., Zhao M., Meng Z. (2017). Expression analysis of genes encoding double B-box zinc finger proteins in maize. Funct. Integr. Genomic..

[38] Bai S., Tao R., Tang Y., Yin L., Ma Y., Ni J., Yan X., Yang Q., Wu Z., Zeng Y. (2019). BBX16, a B-box protein, positively regulates light-induced anthocyanin accumulation by activating MYB10 in red pear. Plant Biotechnol. J..

[39] Shalmani A., Fan S., Jia P., Li G., Muhammad I., Li Y., Sharif R., Dong F., Zuo X., Li K. (2018). Genome identification of B-box gene family members in seven Rosaceae species and their expression analysis in response to flower induction in *Malus domestica*. Molecules.

[40] Cai D., Liu H., Sang N., Huang X. (2017). Identification and characterization of CONSTANS-like (*COL*) gene family in upland cotton (*Gossypium hirsutum* L.). PLoS ONE.

[41] Jin H., Xing M., Cai C., Li S. (2020). B-box Proteins in *Arachis duranensis*: Genome-wide characterization and expression profiles analysis. Agronomy.

[42] Chaurasia A.K., Patil H.B., Azeez A., Subramaniam V.R., Krishna B., Sane A.P., Sane P.V. (2016). Molecular characterization of CONSTANS-Like (*COL*) genes in banana (*Musa acuminata* L. AAA Group, cv. Grand Nain). Physiol. Mol. Biol. Plants.

[43] Wu F., Price B.W., Haider W., Seufferheld G., Nelson R., Hanzawa Y. (2014). Functional and evolutionary characterization of the CONSTANS gene family in short-day photoperiodic flowering in soybean. PLoS One.

[44] Liu C., Tang Q., Cheng C., Xu Y., Yang Z., Dai Z., Su J. (2018). Identification of putative *CONSTANS-like* genes from the *de novo* assembled transcriptome of leek. Biol. Plant..

[45] Chia T.Y., Müller A., Jung C., Mutasa-Göttgens E.S. (2008). Sugar beet contains a large CONSTANS-LIKE gene family including a CO homologue that is independent of the early-bolting (B) gene locus. J. Exp. Bot..

[46] Purugganan M., Fuller D.Q. (2009). The nature of selection during plant domestication. Nature.

[47] Andrés F., Coupland G. (2012). The genetic basis of flowering responses to seasonal cues. Nat. Rev. Genet..

[48] Pajoro A., Biewers S., Dougali E., Valentim F.L., Mendes M.A., Porri A., Coupland G., van de Peer Y., van Dijk A.D., Colombo L. (2014). The (r)evolution of gene regulatory networks controlling Arabidopsis plant reproduction: A two-decade history. J. Exp. Bot..

[49] Shim J.S., Imaizumi T. (2015). Circadian clock and photoperiodic response in Arabidopsis: From seasonal flowering to redox homeostasis. Biochemistry.

[50] Tripathi P., Carvallo M., Hamilton E.E., Preuss S., Kay S.A. (2017). Arabidopsis B-BOX32 interacts with CONSTANS-LIKE3 to regulate flowering. Proc. Natl. Acad. Sci. USA.

[51] Hassidim M., Harir Y., Yakir E., Kron I., Green M.R. (2009). Overexpression of CONSTANS-LIKE 5 can induce flowering in short-day grown Arabidopsis. Planta.

[52] Cheng X.F., Wang Z.Y. (2005). Overexpression of COL9, a CONSTANS-LIKE gene, delays flowering by reducing expression of *CO* and *FT* in *Arabidopsis thaliana*. Plant J..

[53] Ordonez-Herrera N., Trimborn L., Menje M., Henschel M., Robers L., Kaufholdt D., Hansch R., Adrian J., Ponnu J., Hoecker U. (2018). The transcription factor COL12 is a substrate of the COP1/SPA E3 ligase and regulates flowering time and plant architecture. Plant Physiol..

[54] Takase T., Kakikubo Y., Nakasone A., Nishiyama Y., Yasuhara M., Tokioka-Ono Y., Kiyosue T. (2011). Characterization and transgenic study of CONSTANS-LIKE8 (COL8) gene in *Arabidopsis thaliana*: Expression of 35S:COL8 delays flowering under long-day conditions. Plant Biotechnol..

[55] Kojima S., Takahashi Y., Kobayashi Y., Monna L., Sasaki T., Araki T., Yano M. (2002). Hd3a, a rice ortholog of the Arabidopsis *FT* gene, promotes transition to flowering downstream of Hd1 under short-day conditions. Plant Cell Physiol..

[56] Lee Y.S., Jeong D.H., Lee D.Y., Yi J., Ryu C.H., Kim S.L., Jeong H.J., Choi S.C., Jin P., Yang J. (2010). OsCOL4 is a constitutive flowering repressor upstream of Ehd1 and downstream of OsphyB. Plant J..

[57] Liu H., Gu F., Dong S., Liu W., Wang H., Chen Z., Wang J. (2016). CONSTANS-like 9 (COL9) delays the flowering time in *Oryza sativa* by repressing the Ehd1 pathway. Biochem. Biophys. Res. Commun..

[58] Tan J., Jin M., Wang J., Wu F., Sheng P., Cheng Z., Wang J., Zheng X., Chen L., Wang M. (2016). *OsCOL10*, a CONSTANS-like gene, functions as a flowering time repressor downstream of Ghd7 in rice. Plant Cell Physiol..

[59] Sheng P., Wu F., Tan J., Zhang H., Ma W., Chen L., Wang J., Wang J., Zhu S., Guo X. (2016). A CONSTANS-like transcriptional activator, OsCOL13, functions as a negative regulator of flowering downstream of *OsphyB* and upstream of *Ehd1* in rice. Plant Mol. Biol..

[60] Wu W., Zhang Y., Zhang M., Zhan X., Shen X., Yu P., Chen D., Liu Q., Sinumporn S., Hussain K. (2018). The rice CONSTANS-like protein OsCOL15 suppresses flowering by promoting Ghd7 and repressing RID1. Biochem. Biophys. Res. Commun..

[61] Miller T.A., Muslin E.H., Dorweiler J.E. (2008). A maize CONSTANS like gene, *Conz1*, exhibits distinct diurnal expression patterns in varied photoperiods. Planta.

[62] Meng X., Muszynski M.G., Danilevskaya O.N. (2011). The *FT*-Like *ZCN8* gene functions as a floral activator and is involved in photoperiod sensitivity in maize. Plant Cell.

[63] Yang S., Weers B.D., Morishige D.T., Mullet J.E. (2014). *CONSTANS* is a photoperiod regulated activator of flowering in sorghum. BMC Plant Biol..

[64] Yang Y., Ma C., Xu Y., Wei Q., Imtiaz M., Lan H., Gao S., Cheng L., Wang M., Fei Z. (2014). A zinc finger protein regulates flowering time and abiotic stress tolerance in chrysanthemum by modulating gibberellin biosynthesis. Plant Cell.

[65] Campoli C., Drosse B., Searle I., Coupland G., von Korff M. (2012). Functional characterisation of HvCO1, the barley (*Hordeum vulgare*) flowering time ortholog of CONSTANS. Plant J..

[66] Kikuchi R., Kawahigashi H., Oshima M., Ando T., Handa H. (2012). The differential expression of HvCO9, a member of the CONSTANS-like gene family, contributes to the control of flowering under short-day conditions in barley. J. Exp. Bot..

[67] Bai B., Zhao J., Li Y., Zhang F., Zhou J., Chen F., Xie X. (2016). OsBBX14 delays heading date by repressing florigen gene expression under long and short-day conditions in rice. Plant Sci..

[68] Wu W., Zheng X.M., Chen D., Zhang Y., Ma W., Zhang H., Sun L., Yang Z., Zhao C., Zhan X. (2017). OsCOL16, encoding a CONSTANS-like protein, represses flowering by up-regulating *Ghd7* expression in rice. Plant Sci..

[69] Kim S.K., Yun C.H., Lee J.H., Jang Y.H., Park H.Y., Kim J.K. (2008). OsCO3, a CONSTANS-LIKE gene, controls flowering by negatively regulating the expression of *FT*-like genes under SD conditions in rice. Planta.

[70] Ping Q., Cheng P., Huang F., Ren L., Cheng H., Guan Z., Fang W., Chen S., Jiang J. (2019). The heterologous expression in *Arabidopsis thaliana* of a chrysanthemum gene encoding the BBX family transcription factor CmBBX13 delays flowering. Plant Physiol. Biochem..

[71] Wang L., Sun J., Ren L., Zhou M., Han X., Ding L., Zhang F., Guan Z., Fang W., Chen S. (2020). CmBBX8 accelerates flowering by targeting CmFTL1 directly in summer chrysanthemum. Plant Biotechnol. J..

[72] Molesini B., Dusi V., Pennisi F., Di Sansebastiano G.P., Zanzoni S., Manara A., Furini A., Martini F., Rotino G.L., Pandolfini T. (2020). TCMP-2 affects tomato flowering and interacts with BBX16, a homolog of the Arabidopsis B-box MiP1b. Plant Direct..

[73] Abelenda J.A., Cruz-Oró E., Franco-Zorrilla J.M., Prat S. (2016). Potato St CONSTANS-like1 suppresses storage organ formation by directly activating the FT-like StSP5G repressor. Curr. Biol..

[74] Martínez-García J.F., Virgós-Soler A., Prat S. (2002). Control of photoperiod-regulated tuberization in potato by the Arabidopsis flowering-time gene CONSTANS. Proc. Natl. Acad. Sci. USA.

[75] Martin J., Storgaard M., Andersen C.H., Nielsen K.K. (2004). Photoperiodic regulation of flowering in perennial ryegrass involving a *CONSTANS*-like homolog. Plant Mol. Biol..

[76] Almada R., Cabrera N., Casaretto J.A., Ruiz-Lara S., Villanueva E.G. (2009). *VvCO* and *VvCOL1*, two CONSTANS homologous genes, are regulated during flower induction and dormancy in grapevine buds. Plant Cell Rep..

[77] Herrmann D., Barre P., Santoni S., Julier B. (2010). Association of a CONSTANS-LIKE gene to flowering and height in autotetraploid alfalfa. Theor. Appl. Genet..

[78] González-Schain N.D., Díaz-Mendoza M., Zurczak M., Suárez-López P. (2012). Potato CONSTANS is involved in photoperiodic tuberization in a graft-transmissible manner. Plant J..

[79] Ballerini E.S., Kramer E.M. (2011). In the light of evolution: A reevaluation of conservation in the *CO*-*FT* regulon and its role in photoperiodic regulation of flowering time. Front. Plant Sci..

[80] Abelenda J.A., Navarro C., Prat S. (2014). Flowering and tuberization: A tale of two night shades. Trends Plant Sci..

[81] Molesini B., Rotino G.L., Dusi V., Chignola R., Sala T., Mennella G., Francese G., Pandolfini T. (2018). Two metallocarboxypeptidase inhibitors are implicated in tomato fruit development and regulated by the inner no outer transcription factor. Plant Sci..

[82] Chen X.R., Wang Y., Zhao H.H., Zhang X.Y., Wang X.B., Li D.W., Yu J.L., Han C.G. (2018). Brassica yellows virus’ movement protein upregulates anthocyanin accumulation, leading to the development of purple leaf symptoms on *Arabidopsis thaliana*. Sci. Rep..

[83] Li X.L., Lv X., Wang X., Wang L., Zhang M., Ren M. (2018). Effects of abiotic stress on anthocyanin accumulation and grain weight in purple wheat. Crop Pasture Sci..

[84] Zheng X.T., Yu Z.C., Tang J.W., Cai M.L., Chen Y.L., Yang C.W., Chow W.S., Peng C.L. (2020). The major photoprotective role of anthocyanins in leaves of *Arabidopsis thaliana* under long-term high light treatment: Antioxidant or light attenuator?. Photosynth. Res..

[85] Bai S., Saito T., Honda C., Hatsuyama Y., Ito A., Moriguchi T. (2014). An apple B-box protein, MdCOL11, is involved in UV-B- and temperature-induced anthocyanin biosynthesis. Planta.

[86] Plunkett B.J., Henry-Kirk R., Friend A., Diack R., Helbig S., Mouhu K., Tomes S., Dare A.P., Espley R.V., Putterill J. (2019). Apple B-box factors regulate light-responsive anthocyanin biosynthesis genes. Sci. Rep..

[87] Fang H., Dong Y., Yue X., Hu J., Jiang S., Xu H., Wang Y., Su M., Zhang J., Zhang Z. (2019). The B-box zinc finger protein MdBBX20 integrates anthocyanin accumulation in response to ultraviolet radiation and low temperature. Plant Cell Environ..

[88] An J.P., Wang X.F., Zhang X.W., Bi S.Q., You C.X., Hao Y.J. (2019). MdBBX22 regulates UV-B-induced anthocyanin biosynthesis through regulating the function of MdHY5 and is targeted by MdBT2 for 26S proteasome-mediated degradation. Plant Biotech. J..

[89] An J.P., Wang X.F., Espley R.V., Lin-Wang K., Bi S.Q., You C.X., Hao Y.J. (2020). An apple B-Box protein MdBBX37 modulates anthocyanin biosynthesis and hypocotyl elongation synergistically with MdMYBs and MdHY5. Plant Cell Physiol..

[90] Bai S., Tao R., Yin L., Ni J., Yang Q., Yan X., Yang F., Guo X., Li H., Teng Y. (2019). Two B-box proteins, PpBBX18 and PpBBX21, antagonistically regulate anthocyanin biosynthesis via competitive association with *Pyrus pyrifolia* ELONGATED HYPOCOTYL 5 in the peel of pear fruit. Plant J..

[91] Takuhara Y., Kobayashi M., Suzuki S. (2011). Low-temperature-induced transcription factors in grapevine enhance cold tolerance in transgenic Arabidopsis plants. J. Plant Physiol..

[92] Liu X., Li R., Dai Y., Yuan L., Sun Q., Zhang S., Wang X. (2019). A B-box zinc finger protein, *Md*BBX10, enhanced salt and drought stresses tolerance in Arabidopsis. Plant Mol. Biol..

[93] Shalmani A., Jing X.Q., Shi Y., Muhammad I., Zhou M.R., Wei X.Y., Chen Q.Q., Li W.Q., Liu W.T., Chen K.M. (2019). Characterization of B-BOX gene family and their expression profiles under hormonal, abiotic and metal stresses in Poaceae plants. BMC Genomics.

[94] Liu J., Shen J., Xu Y., Li X., Xiao J., Xiong L. (2016). Ghd2, a CONSTANS-like gene, confers drought sensitivity through regulation of senescence in rice. J. Exp. Bot..

[95] Chen J., Chen J.Y., Wang J.N., Kuang J.F., Shan W., Lu W.J. (2012). Molecular characterization and expression profiles of *MaCOL1*, a CONSTANS-like gene in banana fruit. Gene.

[96] Zhang H., Zhang Q., Zhai H., Gao S., Yang L., Wang Z., Xu Y., Huo J., Ren Z., Zhao N. (2020). IbBBX24 promotes the jasmonic acid pathway and enhances fusarium wilt resistance in sweet potato. Plant Cell.

[97] Liu H., Dong S., Sun D., Liu W., Gu F., Liu Y., Guo T., Wang H., Wang J., Chen Z. (2016). CONSTANS-like 9 (OsCOL9) interacts with receptor for activated C-Kinase 1 (OsRACK1) to regulate blast resistance through salicylic acid and ethylene signaling pathways. PLoS ONE.

[98] Vaishak K.P., Yadukrishnan P., Bakshi S., Kushwaha A.K., Ramachandran H., Job N., Babu D., Datta S. (2019). The B-box bridge between light and hormones in plants. J. Photochem. Photobiol. B..

[99] Hou H., Jia H., Yan Q., Wang X. (2018). Overexpression of a SBP-Box Gene (*VpSBP16*) from chinese wild vitis species in Arabidopsis improves salinity and drought stress tolerance. Int. J. Mol. Sci..

[100] An J.P., Wang X.F., Zhang X.W., You C.X., Hao Y.J. (2021). Apple B-box protein BBX37 regulates jasmonic acid mediated cold tolerance through the JAZ-BBX37-ICE1-CBF pathway and undergoes MIEL1-mediated ubiquitination and degradation. New Phytol..

[101] Mbambalala N., Panda S.K., van der Vyver C. (2020). Overexpression of *AtBBX29* improves drought tolerance by maintaining photosynthesis and enhancing the antioxidant and osmolyte capacity of sugarcane plants. Plant Mol. Biol. Rep..

[102] Kiełbowicz-Matuk A., Czarnecka J. (2014). Interplays of plant circadian clock and abiotic stress response network. Emerging Technologies and Management of Crop Stress Tolerance, Volume 1-Biological Techniques.

[103] Bendix C., Marshall C.M., Harmon F.G. (2015). Circadian clock genes universally control key agricultural traits. Mol. Plant.

[104] Piechulla B., Merforth N., Rudolph B. (1998). Identification of tomato Lhc promoter regions necessary for circadian expression. Plant Mol. Biol..

[105] Kiełbowicz-Matuk A., Czarnecka J., Banachowicz E., Rey P., Rorat T. (2017). *Solanum tuberosum* ZPR1 encodes a light-regulated nuclear DNA-binding protein adjusting the circadian expression of *StBBX24* to light cycle. Plant Cell Environ..

[106] Song Z., Bian Y., Liu J., Sun Y., Xu D. (2020). B-box proteins: Pivotal players in light-mediated development in plants. J. Integr. Plant Biol..

